# Simultaneous Bilateral Ophthalmic Artery Chemosurgery for Bilateral Retinoblastoma (Tandem Therapy)

**DOI:** 10.1371/journal.pone.0156806

**Published:** 2016-06-03

**Authors:** David H. Abramson, Brian P. Marr, Jasmine H. Francis, Ira J. Dunkel, Armida W. M. Fabius, Scott E. Brodie, Ijah Mondesire-Crump, Y. Pierre Gobin

**Affiliations:** 1 Department of Surgery, Memorial Sloan Kettering Cancer Center, New York, New York, United States of America; 2 Department of Ophthalmology, Weill Cornell Medical College, New York Presbyterian Hospital, New York, New York, United States of America; 3 Department of Pediatrics, Memorial Sloan Kettering Cancer Center, New York, New York, United States of America; 4 Department of Pediatrics, Weill Cornell Medical College, New York Presbyterian Hospital, New York, New York, United States of America; 5 Department of Ophthalmology, VU University Medical Center, Amsterdam, Netherlands; 6 Department of Ophthalmology, Icahn School of Medicine at Mount Sinai, New York, New York, United States of America; 7 Department of Radiology, Weill Cornell Medical College, New York Presbyterian Hospital, New York, New York, United States of America; Massachusetts Eye & Ear Infirmary, Harvard Medical School, UNITED STATES

## Abstract

**Objective:**

Report on the 7-year experience with bilateral ophthalmic artery chemosurgery (OAC-Tandem therapy) for bilateral retinoblastoma.

**Design:**

Retrospective, single institution study.

**Subjects:**

120 eyes of 60 children with bilateral retinoblastoma treated since March 2008.

**Methods:**

Retrospective review of all children treated at Memorial Sloan Kettering with bilateral ophthalmic artery chemosurgery (Melphalan, Carboplatin, Topotecan, Methotrexate) delivered in the same initial session to both naïve and previously treated eyes.

**Main Outcome Measures:**

Ocular survival, metastatic disease, patient survival from metastases, second cancers, systemic adverse effects, need for transfusion of blood products, electroretinogram before and after treatment.

**Results:**

116 eyes were salvaged (4 eyes were enucleated: 3 because of progressive disease, 1 family choice). Kaplan Meier ocular survival was 99.2% at one year, 96.9% at 2 and 3 years and 94.9% for years 4 through 7. There were no cases of metastatic disease or metastatic deaths with a mean follow-up of 3.01 years. Two children developed second cancers (both pineoblastoma) and one of them died. Transfusion of blood products was required in 3 cases (4 transfusions), 1.9%. Two children developed fever/neutropenia requiring hospitalization (0.95%). ERGs were improved in 21.6% and unchanged after treatment in 52.5% of cases (increase or decrease of less than 25μV).

**Conclusions:**

Bilateral ophthalmic artery chemosurgery is a safe and effective technique for managing bilateral retinoblastoma-even when eyes are advanced bilaterally, and if both eyes have progressed after systemic chemotherapy. Ocular survival was excellent (94.9% at 8 years), there were no cases of of metastatic disease and no deaths from metastatic disease, but children remain at risk for second cancers. In 21.6% of cases ERG function improved. Despite using chemotherapy in both eyes in the same session, systemic toxicity was low.

## Introduction

Ophthalmic artery chemosurgery (OAC) was introduced almost 10 years ago for the treatment of retinoblastoma[1] and has been performed in more than 45 countries worldwide for unilateral retinoblastoma.[2] In a recent survey it was the first-line choice for the majority of retinoblastoma centers worldwide for patients with advanced unilateral disease.[3] The technique has been successful for naïve eyes[4,5] and eyes that failed all prior therapies.[6,7] It has enabled the majority of advanced eyes that were previously managed with enucleation to be saved–[4,8–10] sometimes with useful vision–[10,11] without compromising patient survival.[11,12] In addition to saving eyes with advanced intraocular disease[8,9] it has been used for eyes with less advanced disease,[10,11] eyes with vitreous seeding, sub-retinal seeding [13,14] and as salvage therapy in eyes that progressed after a prior course of intrarterial chemotherapy.[14,15] It has also been shown to significantly reduce the development of new intraocular tumor foci in germline cases. [15,16]

OAC has been used for one eye in bilateral cases by many centers after one eye was enucleated or in cases of asymmetric bilateral disease (allowing focal treatments only for one eye and OAC for the fellow eye) but the first report on *simultaneous* treatment of both eyes (called “tandem therapy”) was published just 5 years ago.[12] Since that initial report there have been more than 200 peer review publications on the indications, results, complications and other issues in *unilateral* cases but no additional case series employing simultaneous use for bilateral disease. Fewer than 10 such cases have been reported in the literature since our description of the technique.

We now have experience with more than 200 bilateral cases and more than 60 bilateral cases treated simultaneously so we performed a single institution retrospective analysis of all bilateral cases where both eyes were treated in the same session to determine patient survival, systemic complications, ocular survival and ocular complications.

## Methods

This is a retrospective chart review, approved by the Memorial Sloan Kettering Cancer Center (MSKCC) Institutional Review Board (IRB), of all bilateral retinoblastoma patients whose initial management was simultaneous bilateral OAC at MSKCC between March 2008 and May 2015. Bilateral patients who received OAC in one eye and non-OAC treatments in the fellow eye were not included. Patients who had previously been treated elsewhere with OAC and then received simultaneous OAC at our institution were not included, but any patient who had prior therapy that did not include OAC was included so long as the first treatment at MSKCC was with tandem therapy. Clinical characteristics including laterality, treatment parameters, sex, age at diagnosis, electroretinography (ERG) before and after treatment, ocular and patient survival and second tumors were collected. Patient information was anonymized and deidentified prior to analysis.

We have previously described our tandem technique[16,17] though there have been some refinements in technique since our initial report 5 years ago. A microcatheter is inserted into the femoral artery and passed through the abdominal and thoracic aorta into the internal carotid artery on one side. After advancing the catheter to the orifice of the ophthalmic artery, chemotherapy is infused. The catheter is then withdrawn to the aorta and advanced up the contralateral side to the orifice of the fellow ophthalmic artery for the second chemotherapy infusion. At the end of the procedure the microcatheter is removed, manual pressure is applied, and the patients are observed for 4–6 hours before being discharged the same day.

Catheterization of the ophthalmic artery is performed with a microcatheter only- a guide catheter is no longer used. When treating both eyes we deliver all drug(s) within a one hour time period. Nasal vasoconstrictors and topical phenylephrine on the skin in the distribution of the supratrochlear artery were routinely used.

Electroretinograms (ERGs) were performed before and at the follow up visits 3–4 weeks after each treatment. Measurements are taken immediately following anesthetic induction but before the ocular exam; scleral indentations affect recorded potentials.[18]

### Electroretinography

As previously reported we adapted the International Society for Clinical Electrophysiology of Vision’s standard ERG protocol to obtain electroretinography (ERG) recordings.[19] The 30-Hz photopic flicker amplitude data were used, which we have previously reported to be an accurate representation of the complete ERG response protocol.[20] The initial ERG was compared to the most recent ERG. 30-Hz response amplitude changes of >25 μV were considered clinically significant. As in our earlier published series ERG amplitudes were capped at 100.1 μV. ERG amplitudes were classified as follows: less than 0.1 μV, undetectable; 0.1–25 μV, poor; 25.1–50 μV, fair; 50.1–75 μV, good; 75.1–100 μV very good; more than 100 μV, excellent.

## Results

Patient Characteristics are presented in Table 1. Ocular Characteristics of treated eyes are presented in Table 2. OAC treatment details are presented in Tables 3 & 4. Of the 120 eyes treated 13 subsequently received either an additional course(s) of OAC or radiation (7 cases received additional OAC, 6 received a plaque, and 1 received external beam radiation at another Institution).

**Table 1:** Patient Demographics.

**Gender**	Male:	26	
	Female:	34	
	Total:	60	
**Family History**	Neg:	48	
	Pos:	11	
	Unkn:	1	
	Total:	60	
	Mean	Median	Range
**Age at Presentation** (yrs)	0.91	0.71	0.16—4.46
**Patient Follow up** (yrs)	3.12	3.05	0.18—7.12

**Table 2:** Eye Classifications & Group Salvage.

International Classification (ICRb)			Reese Ellsworth (RE)		
Group	Number of Eyes (% of total)	Eyes Salvaged (% salvaged)	Group	Number of Eyes (% of total)	Eyes Salvaged (% salvaged)
**A:**	2 (1.7)	2 (100)	**I:**	3 (2.5)	3 (100)
**B:**	18 (15.0)	18 (100)	**II:**	9 (7.5)	9 (100)
**C:**	24 (20.0)	24 (100)	**III:**	19 (15.8)	19 (100)
**D:**	56 (46.7)	54 (100)	**IV:**	12 (10.0)	12 (100)
**E:**	20 (16.7)	18 (90.0)	**V:**	77 (64.2)	73 (94.8)
**Total:**	120 (100)	116/120 (96.7)	**Total:**	120 (100)	116/120 (96.7)

**Table 3:** OAC Treatment Details.

**OAC Treatments**	
Total (per eye)	418
Mean (per eye)	3.48
Range	1.0–8.0
OAC Sessions*	
Total	256
Mean (per patient)	4.27
Range	1.0–11.0
**Tandem Therapy**	
Total	156.0
Mean (per patient)	1.3
Range	1.0–6.0

*Session defined as either unilateral or tandem OAC following an anesthetic induction.

**Table 4:** Drug Combinations Used Per OAC Treatment.

Chemotherapy	Number of treatments (#)	Percent of total (%)
M+T	101	24.3
M	98	23.6
T+C	79	19.0
M+T+C	58	14.0
C	51	12.3
M+C	25	6.0
T	2	0.5
Mtx	1	0.2
Total*:	415	

M = Melphalan, T = Topotecan, C = Carboplatin, Mtx = Methotrexate

*In 3 cases the OAC procedure was terminated prior to the administration of chemotherapy.

The distribution of ICRb in the two eyes was: A&B: 1, A&E: 1, B&B: 2, B&C: 3, B&D: 5, B&E: 5, C&C: 3, C&D: 14, C&E: 1, D&D: 14, D&E: 9, E&E: 2.

Half (30) of the patients were naïve to treatment and half (30) received OAC after having been treated with systemic chemotherapy and/or radiation. No patient developed metastatic disease and no patient died from metastatic disease. Two patients developed second cancers (both pineoblastomas) and one died.

**Fig 1:**
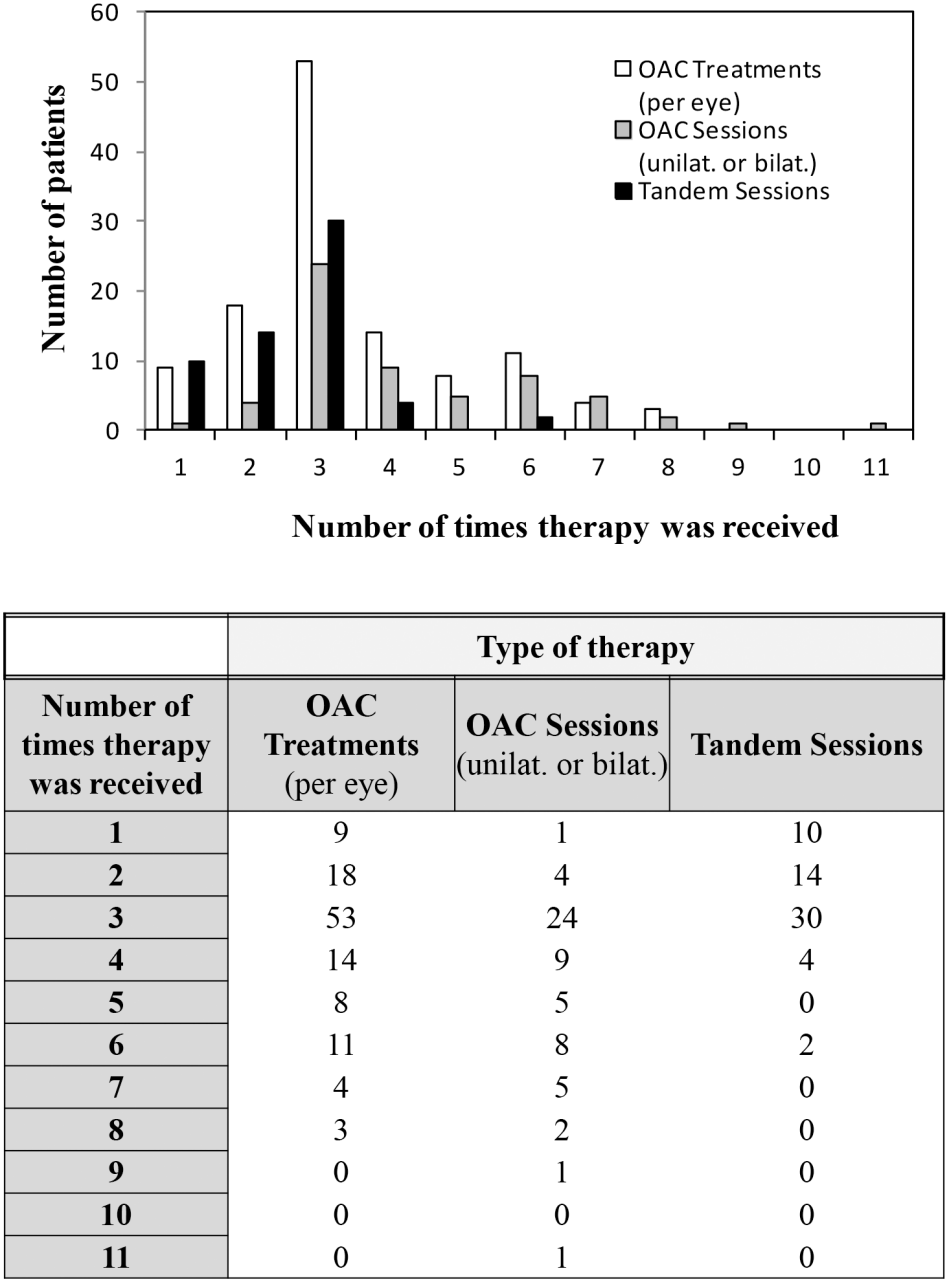
Type and amount of OAC therapy received per number of patients.

One hundred twenty eyes were treated in a total of 418 sessions, with the total number of treatments per patient ranging from 1 to 11 (mean 3.46 per patient). Tables 3 & 4 and Fig 1 summarize the OAC treatment details.

**Fig 2:**
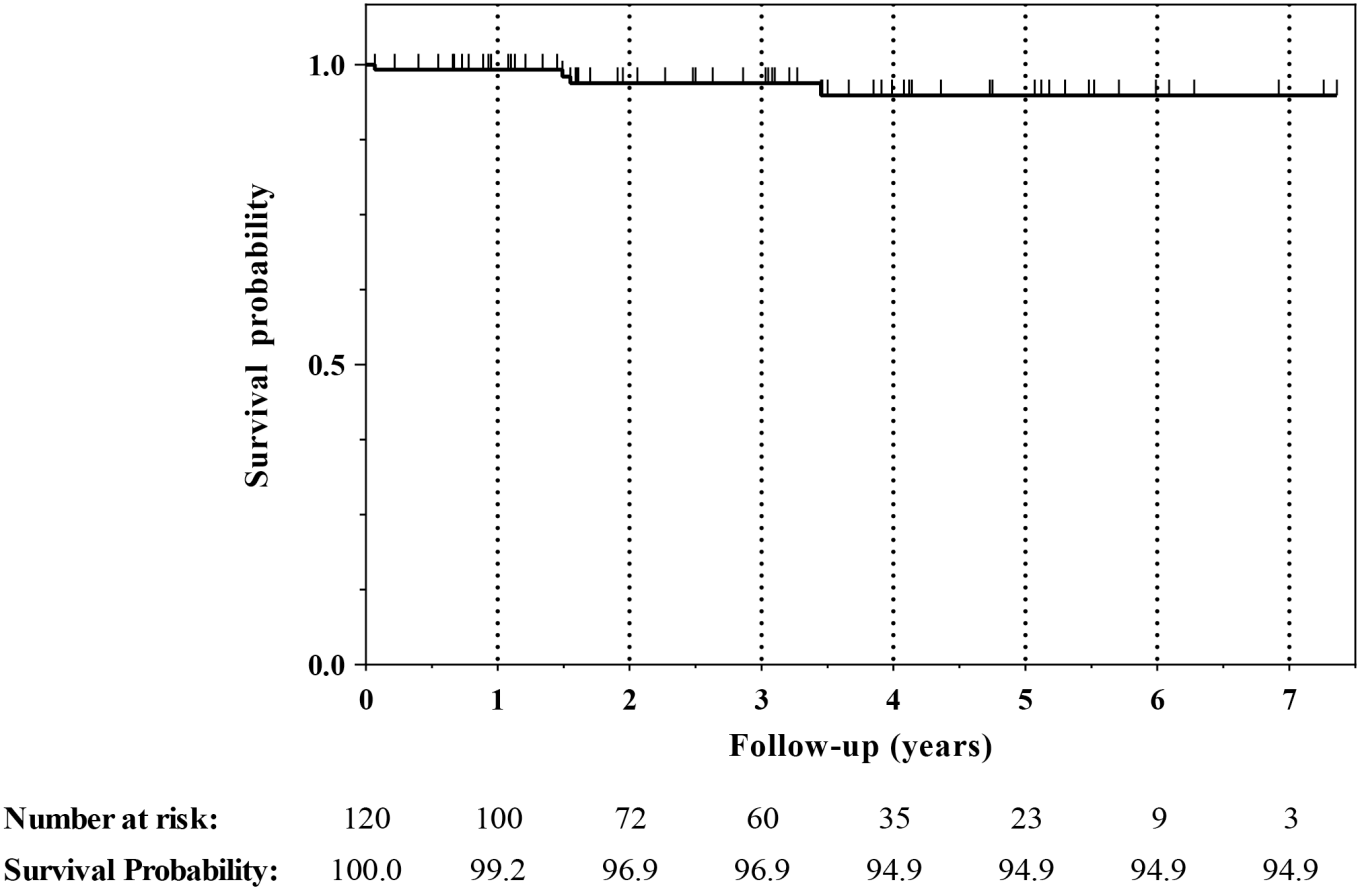
Kaplan Meier Ocular Survival Curve. One hundred and twenty eyes of 60 patients who underwent bilateral ophthalmic artery chemosurgery (Tandem Therapy) were assessed for enucleation. Ocular survival was 99.2% at one year, 96.9% at 2 and 3 years, and 94.9% for years 4 through 7.

Four of the 120 eyes came to enucleation (3 with progressive disease, 1 family choice), and the Kaplan Meier ocular survival rates are presented in Fig 2. There were no bilateral enucleations. The classification of the enucleated eyes was Va/E:1, Vb/E:1, Vb/D:2. There were no procedure deaths or strokes (1 patient had a TIA). The ERG remained the same before and after treatment in 52.5% of eyes while 21.7% improved and 25.8% decreased.

There were 2 cases of fever/neutropenia (0.96%) and 3 cases (4 transfusions) of blood products required (1.9%). 1 patient was admitted for fever/neutropenia without transfusion. 1 patient was admitted twice for fever/neutropenia following two subsequent OAC sessions, receiving a transfusion during each admission. 2 patients received transfusion without admission.

**Fig 3:**
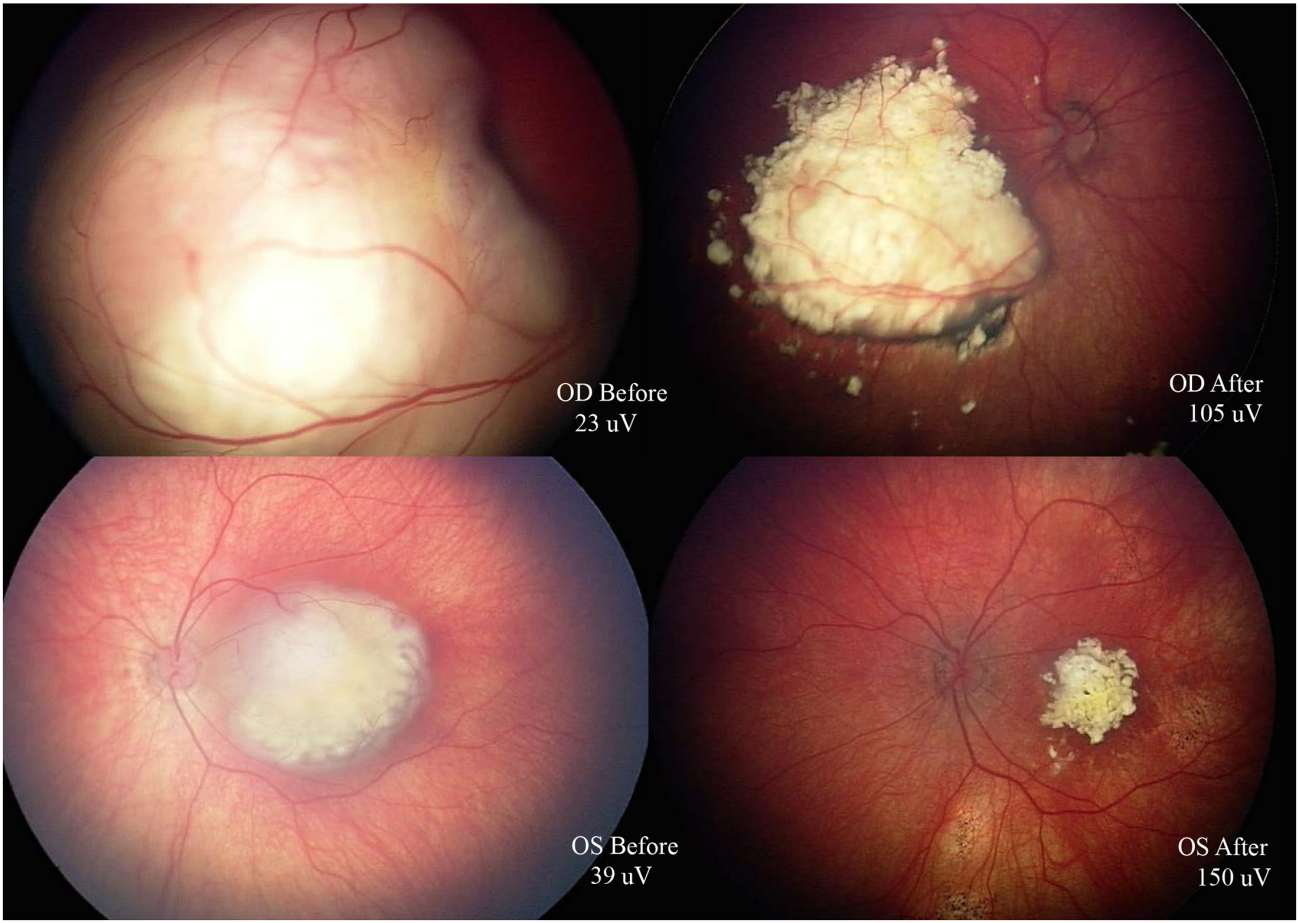
Fundus images from patient before (left) and after (right) tandem OAC. Upper row: right eye, lower row: left eye. ERG Right eye: before 23uV, after 105uV. ERG Left eye: before 39uV, after 150uV.

Fig 3 shows both eyes of one patient before and after treatment with tandem OAC.

## Discussion

Intrarterial chemotherapy has transformed the management of unilateral retinoblastoma worldwide.[2] It has enabled eyes that were previously enucleated worldwide to be salvaged without sacrificing patient survival.[13] Writing in the 1960’s Reese and Duke-Elder emphasized that all unilateral eyes should be enucleated because there was no other therapeutic option available (at the time).[2] In 1982 we published the first large report on the successful treatment of unilateral retinoblastoma without enucleation and demonstrated that survival was not compromised despite trying to save the eye. However, only two of the patients in that series had advanced intraocular disease; they were treated with external beam irradiation.[14]

Bilateral retinoblastoma has been treated with radiation for >80 years. Initially patients were cured but because of radiation damage no eye was salvaged with useful vision. As doses were lowered and techniques improved clinicians would enucleate the “more advanced” eye and irradiate the “less advanced eye” so bilateral simultaneous treatment was unusual.[15] With even more experience and different equipment bilateral simultaneous radiation was successfully done for bilateral retinoblastoma.[16] In cases of bilateral Reese Ellsworth groups I-III success was high. Eighty six per-cent of the eyes were saved and only 5% of the patients died of metastatic retinoblastoma. In select cases bilateral (Reese-Ellsworth groups IV and V) advanced retinoblastoma was also primarily bilaterally radiated. Ocular survival rates were 53% at ten years. Death from metastatic disease was rare but 52.6% of patient developed a second cancer by 18 ¼ years.[17] Largely because of second-cancer development, external beam irradiation was abandoned in favor of multiagent systemic chemotherapy 25 years ago.[21,22] Unfortunately, results with systemic chemotherapy for advanced eyes were disappointing. Virtually all “E” eyes were still enucleated[23] and the majority of “D” eyes were either enucleated or progressed despite systemic chemotherapy.[24] As a result there are few reported successes using systemic chemotherapy for bilateral *advanced* intraocular disease.

In our initial paper on OAC, 100% of the eyes were advanced, scheduled for enucleation and patients had only *unilateral* disease.[1] Five years ago we were the first to report on simultaneous bilateral OAC treatment and named the technique “tandem therapy”.[12] In those 4 patients (8 eyes) results were encouraging and no patient died. Since then we have had considerable experience in managing bilateral retinoblastoma with OAC. Many of our cases of bilateral disease were managed elsewhere and then referred after failing therapy (including, but not limited to, enucleation of one eye). Often the second, only remaining eye was scheduled elsewhere for enucleation. Similarly, naïve bilateral patients seen and managed initially by us who required either enucleation of one eye (rare nowadays) or only focal treatments in one eye were not included in this series.

There are special considerations for patients treated with “tandem therapy”. Melphalan in both humans[25] and animals[26] causes significant bone marrow suppression when total body dose exceeds 0.4mg/kg. In advanced cases 0.4mg/kg is close to the dose we use to treat **one** eye, and if we were to give double the dose, the bone marrow suppression would be profound. As a result, in tandem therapy we treat one eye with Melphalan (not exceeding 0.4mg/kg) and the fellow eye with Carboplatin. Carboplatin is well known to be active against retinoblastoma when delivered intravenously,[27–30] periocularly[31–33] or intra-arterially.[34] Topotecan is active against retinoblastoma in pre- clinical studies[35] and used in all three of these routes when treating retinoblastoma.[36,37] Theoretically, the effect of a topoisomerase inhibitor (Topotecan) used in conjunction with an alkylator (Melphalan or Carboplatin) are additive, so we have used Topotecan in conjunction with both Melphalan and Carboplatin in tandem therapy. Head- to-head randomized trials comparing each of these three drugs to each other have not been done. Like the Japanese,[38] our experience has been that in the doses we are using Melphalan is the most potent of the three drugs. We have used this observation to guide the decision about which eye will initially receive Melphalan or Carboplatin. Often Melphalan is given to the eye with more advanced disease, though at times it has initially been given to the eye with better potential vision. The decision about changing drug combinations on subsequent sessions is a clinical one and does not follow a rigid algorithm, but commonly the drugs are switched in the second or third session so that the Melphalan eye then receives Carboplatin based therapy.

Despite using multiple chemotherapeutic agents in the same patient at the same sitting, few systemic complications have developed. Transfusion of blood product was limited to 4 transfusions in three patients (1.9% of the events) and clinically significant fever/neutropenia occurred in 2 cases (0.96%).

Ocular toxicity paralleled our previous reports on unilateral retinoblastoma managed with OAC[39] and in most cases the ERG was unaffected by treatment. It is notable that in more than 20% of the eyes there was measurable improvement in ERG function.

Despite the fact that the majority of eyes treated with tandem therapy had advanced intraocular disease, no patient has developed metastatic disease and no patient has died of metastasis. Simultaneously treating both eyes did not compromise patient survival. Equally striking is the observation that of 120 eyes treated, only four came to enucleation (one due to family preference). The 5-year Kaplan-Meier ocular survival rate was 94.9%, which is noteworthy since more than half of these patients had very advanced intraocular disease (ICRb D or E) and half had previously received systemic chemotherapy.

Tandem therapy was equally effective for patient and ocular survival if the patient was naive or had previously received global therapy.

Among children in the first 5 years of life the most common second cancer to develop in bilateral cases is pineoblastoma. Two of our patients developed this tumor and one patient died as a result. These numbers are consistent with the latest published meta-analysis on the incidence of trilateral retinoblastoma in bilateral patients who do not receive radiation.[40] In the long term follow-up of the Japanese experience with selective ophthalmic artery infusion for retinoblastoma, pineoblastomas also developed but the authors felt the incidence was not increased when they compared the Japanese patients who had and had not received intrarterial chemotherapy.[41]

In a recent paper on the management of retinoblastoma from four leading centers worldwide there was complete agreement that OAC was first line choice for advanced unilateral retinoblastoma.[13] There was also complete agreement in the management of bilateral retinoblastoma when there was less advanced disease but there was disagreement about the management of bilateral advanced retinoblastoma. Two centers preferred tandem therapy as initial choice but two others felt systemic chemotherapy should be tried first and tandem therapy reserved for eyes that failed or progressed following systemic chemotherapy. The present study gives some assurance to clinicians that tandem therapy can be successful without significant ocular or systemic side effects and without compromising patient survival.
